# Community composting strategies for biowaste treatment: methodology, bulking agent and compost quality

**DOI:** 10.1007/s11356-023-25564-x

**Published:** 2023-02-23

**Authors:** David Alves, Iria Villar, Salustiano Mato

**Affiliations:** Environmental Biology Group, Universidade de Vigo, 36310 Vigo, Spain

**Keywords:** Municipal solid waste, Composting, Bulking agent, Recycling, Decentralised waste management

## Abstract

The European Union’s commitment to increase recycling and recovery rates of municipal solid waste requires significant changes in current waste management. Local governments are developing various strategies for treating the organic fraction of municipal waste (biowaste) via composting. Community composting centres (CCC), green waste collection, treatment points and community gardens are some of these new approaches. Population density and spatial distribution, together with the existence of community green areas, determine the location of the various infrastructures for recycling local biowaste. The composting process consumes high amounts of bulking agent (BA) necessary to provide the structure that allows, amongst other uses, biowaste aeration and microbial surface colonisation. Shredded green waste from parks, gardens and households can be used as BA in community composting and home composting. In this study, a total of 46 compost samples obtained from CCC with two types of handling were analysed: 22 samples treated by vertical flow (VF) and 24 samples treated by horizontal flow (HF). The HF model allowed better use of the volume of modular composting units and the VF model required less effort and time for the CCC operator. Mature, stable and high-nutrient-content composts were obtained with both models. These composts met the legal requirements to be used as an organic amendment, and they can be delivered to the participants or used in community gardens in the municipality.

## Introduction

The production and composition of municipal solid waste have changed throughout human history. Waste generation is expected to increase significantly in the coming years due to economic development, population growth and urban expansion (Li et al. 2013; Adhikari et al. 2010). For this reason, the European Union approved the modification of Directive 2008/98/EC in which separate collection and treatment of municipal biowaste was stipulated (Directive (EU) 2018/851). This directive defines biowaste as biodegradable garden and park waste, food and kitchen waste from households, restaurants, caterers and retail premises, and comparable waste from food processing plants. Biowaste accounts for more than 34% of the municipal solid waste generated, amounting to 86 million tonnes in 2017 in the EU-28 (Brusselaers and Van Der Linden 2020). Directive (EU) 2018/851 established that by 2025, the preparing for re-use and the recycling of municipal waste shall be increased to a minimum of 55% by weight, progressively increasing this percentage up to 65% by weight by 2035. So, these objectives cannot be met without addressing the biowaste fraction of the municipal waste. Biowaste degrades rapidly and has a high nutrient content, so biological treatments are suitable option, and thus, Member States are taking measures to encourage them. Biological treatments, such as anaerobic digestion, vermicomposting and composting, are the most widely used techniques. Anaerobic digestion to obtain biogas is one of the most widely used alternatives in biowaste treatment. However, in order to carry it out effectively, it is necessary to evaluate several chemical parameters of the biowaste (Zamri et al. 2021). Anaerobic digestion has a higher cost than composting (Colvero et al. 2020), but it can be economically more advantageous, depending on plant scale and valorisation of end products (Lin et al. 2019). Vermicomposting of municipal solid waste is currently gaining popularity because it adds value to the waste whilst also reducing volume (Kundariya et al. 2021). However, as Lohri et al. (2017) have stated, not all household biowaste can be treated with vermicomposting due to the intolerance of earthworms to feed on and develop in waste with a high content of meat, fish, grease and oils, amongst others. Composting is a process that can be developed through a simple, low-cost technology that allows biodegradable materials to be transformed into biologically stable materials called compost (Manu et al. 2019; Pai et al. 2019). Compost can be used as a soil amendment and/or fertiliser and as a substrate for plant growth (Komilis and Tziouvaras 2009; Li et al. 2013), so value-added products are obtained from biowaste composting. It reduces the environmental impact of biowaste, recycling of nutrients in the soil is possible and progress is made towards the circular economy (Keng et al. 2020). Segregation, collection and treatment of biowaste at source is not a new system and has been implemented in some countries. Bruni et al. (2020) highlight that in Italian municipalities about 6 million tonnes of food and garden waste are collected to be treated with biological techniques such as composting. Taking into account these reasons, composting stands out as a simple, low-cost recovery technology that allows all types of biowaste and organic by-products to be transformed into products with fertilising capacity (Bruni et al. 2020).

Another new aspect of the European Directive is the commitment to the “Principle of Proximity”, according to which waste should be recycled as close as possible to the source of generation, avoiding collection and transport costs and their associated environmental impacts (Directive (EU) 2018/851). In this way, the treatment of biowaste is promoted at the local or regional level through decentralised composting as opposed to treatment at the regional level, meaning centralised industrial composting. Countries with a very high population density, such as India, have been promoting decentralised composting in rural areas for this type of waste for several years (Zurbrügg et al. 2004). However, institutions such as universities (Keng et al. 2020; Torrijos et al. 2021), and even major cities, such as Chicago (Pai et al. 2019), where large amounts of biowaste are produced, have also developed decentralised composting of biowaste. Since local systems have significantly lower costs than centralised models, decentralised composting also has positive benefits for the soil, nutrient recycling and job creation compared with incineration treatment (Weidner et al. 2020). Decentralised composting includes the separation at source and on-site treatment through home composting (HC), carried out in the producers’ own homes, and community composting (CC), for the joint treatment of several neighbours’ biowaste in a shared location very close to the homes. Adhikari et al. (2010) described the economic benefits of HC and CC for European and Canadian populations compared with landfill and highlighted the need to address the quality of compost to successfully implement on-site composting.

Other municipal biowaste is the green remains consisting of leaves, wood cuttings from pruning and grass collected from parks and gardens of public and private entities. The increase in green areas in population centre and the expansion of cities have led to an increase in this type of waste, which makes its management and treatment necessary (Eades et al. 2020). The physical and chemical characteristics of these types of waste make them good bulking agents (BA) to mix with household food and kitchen waste (Arrigoni et al. 2018; Reyes-Torres et al. 2018). BA are commonly fibrous with carboneous material, such as agricultural, forestry and other green waste. BA are used to provide air space in composting materials, regulate the water content and the C/N ratio (Iqbal et al. 2010). There are some research papers in which the capacity of different BA for biowaste composting is evaluated and in which their physical properties are highlighted: improvement of the porosity and the FAS (percentage of free air space) of the mixture (Chang and Chen 2010; Iqbal et al. 2010; López et al. 2010; Schwalb et al. 2011).

This paper describes the development and location of community composting centres (CCC) in various municipalities in the Autonomous Community of Galicia (Spain). The importance of the use of green waste as BA in the treatment of the municipal organic fraction for composting is also discussed. The compost obtained in the CCC through vertical and horizontal treatment systems is analysed and classified according to Spanish regulations for use as fertiliser in community gardens and/or recycled in agricultural plots in the same locality, avoiding the transport of waste outside the municipality.

## Material and methods

### Study area

**Fig. 1:**
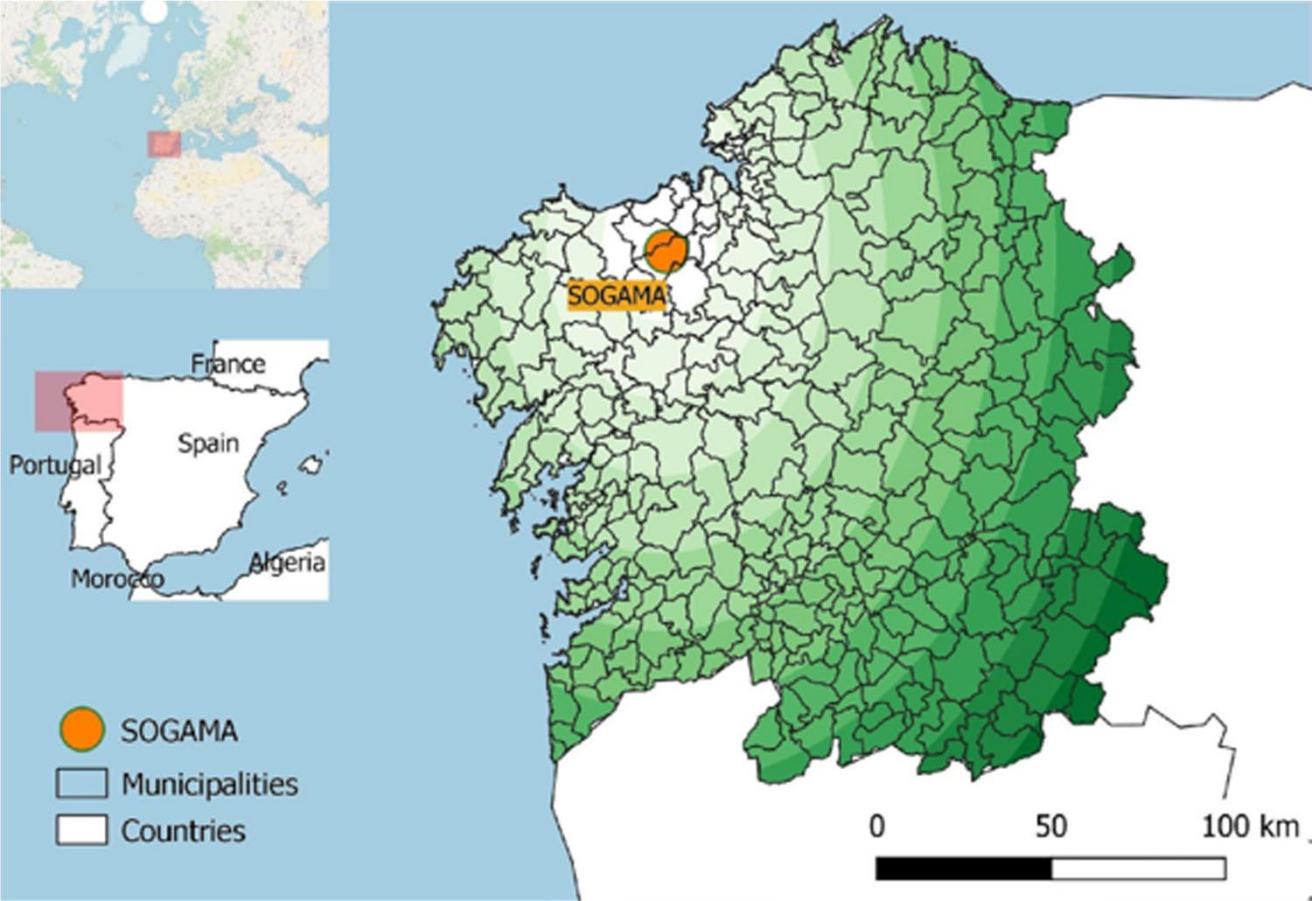
Geographical location of the study area. Spain and Galicia detail showing the municipal boundaries and the SOGAMA facility inset by an orange circle

The Autonomous Community of Galicia, located in the northwest of Spain, has a total area of 29,574 km^2^ and a population of around 2.7 million inhabitants. With a population density of 92 inhabitants/km^2^, the most urbanised areas are mainly concentrated on the coast, whilst dispersed rural population centres are inland and in the east of the region. In Galicia, there are three different municipal waste collection systems (Xunta 2011), including the model of the Galician Environmental Company (Sociedade Galega do Medio Ambiente, S.A. (SOGAMA)). SOGAMA is a public company responsible for managing the municipal solid waste generated by the residents of 295 Galicia municipalities, more than 80% of the region’s population. SOGAMA’s treatment plant receives the waste deposited in the yellow bin for lightweight packaging (plastic, metal and liquid packaging board) and the bin for the mixed fraction, i.e. all waste not collected separately, in this case, biowaste together with sanitary textiles, ceramic waste, household cleaning waste, etc. As can be seen in Fig. 1, waste can travel more than 150 km before it is managed in the treatment plant in the northwest of the region. The biodegradable fraction, which accounts for about 42% of the total amount of waste generated in homes (Xunta 2011), is not collected separately and is segregated together with the waste deposited in the mixed bin. Therefore, the end-use of the biowaste, once it reaches the treatment plant, is incineration for energy recovery. The complexity and heterogeneity of the biowaste deposited by citizens create great variability in its composition although it is characterised by high moisture and, therefore, a low calorific value (Pham et al. 2015). Furthermore, as pointed out by Di Maria et al. (2021), the incineration of this waste increases the negative impacts on the environment and human health of the gases and particles emitted during this process. Recently, SOGAMA’s model has incorporated a new biowaste composting facility, with a capacity of 15 × 10^3^ tonnes per year, and the regional government plans to build several composting plants at the county level. Also, local governments are implementing different biowaste management models.

### Decentralised treatment and CCC methodology

Of all the municipal waste produced, biowaste is the only one for which it is possible to carry out stabilisation and recycling treatment on site through biological techniques such as composting. For this reason, the regulations issued by the European Union insist upon the use of composting at the local level as a method to avoid the transport of waste over long distances and boost the local economy, producing great environmental improvements, reducing the ecological footprint and fighting against climate change (Directive (EU) 2018/851).

**Fig. 2:**
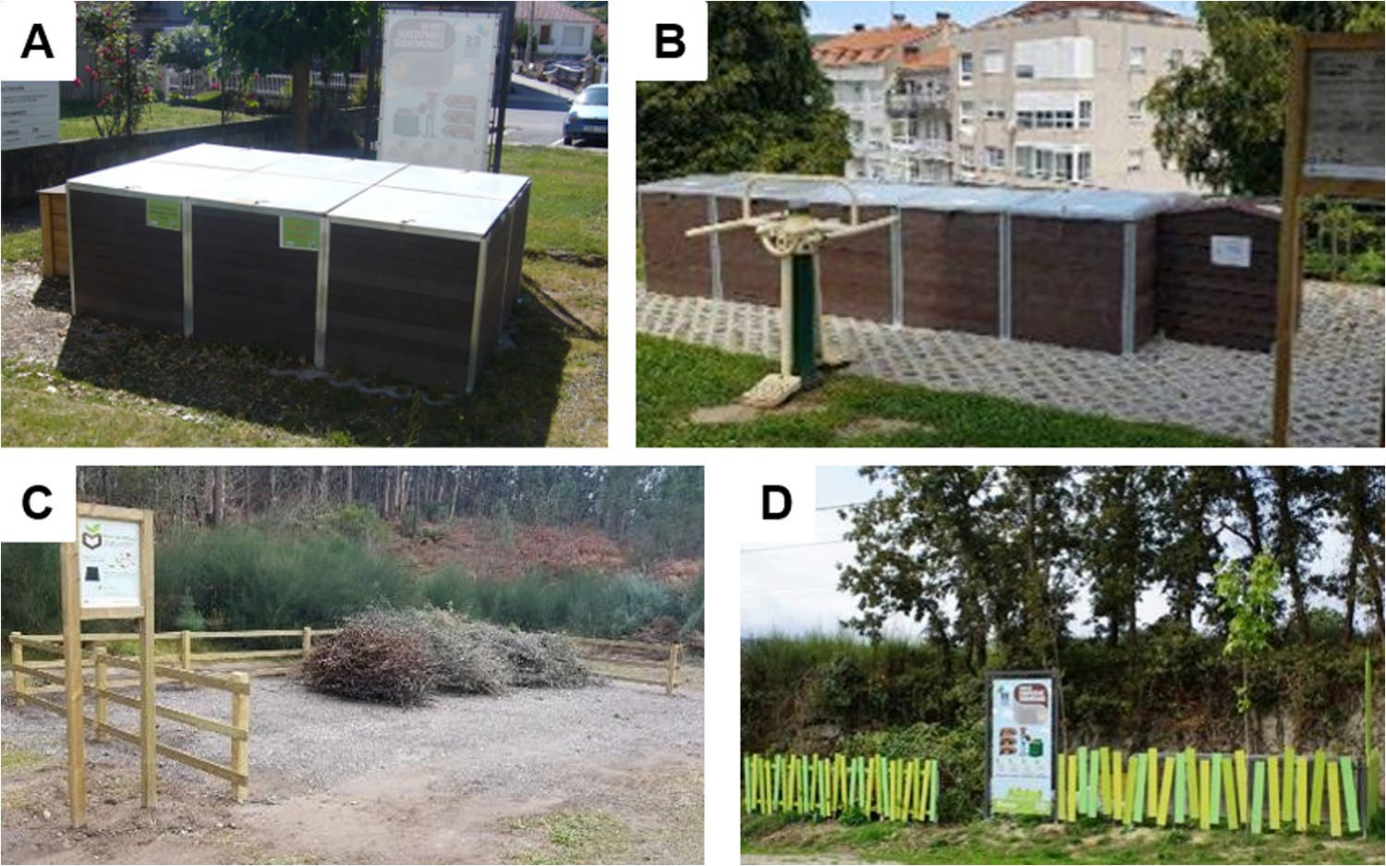
**a**, **b** Community composting centres (CCC); **c**, **d** green waste collection points

Decentralised composting can be carried out through HC and CC depending on various factors such as population density, type of housing and the existence of community green areas (Bruni et al. 2020). CCC are facilities where the biowaste generated by a community of residents or the producers in a built-up area is treated by composting. Some businesses with limited biowaste production can also make use of this type of community system. In addition, in many cases, these systems are open to the public, so the surrounding population, visitors and commuters can also use them. This close proximity to producers means that CCC are considered on-site treatment systems (Adhikari et al. 2010). Currently, the progress and importance of such infrastructure have led several public bodies to develop specific legislation (Pontevedra Provincial Council 2019; Basque Government 2019) and publish CCC work protocols (Fertile Auro 2019). These CCC can be made up of modular composting units where the different phases of composting are carried out (Fig. 2), or they can be established through the provision of individual composters with a greater capacity than those used in a single-family home. The composting process in CCC can be carried out in a similar way to a home composter or vertical flow model (VF) or by separating the process phases into different treatment modules or the horizontal flow model (HF) (Mato et al. 2019). In the HF model, composting is carried out in different consecutive modules. It is necessary to differentiate between the supply module (module 1) for fresh biowaste input, the homogenisation module (module 2), and the maturation module (module 3). CCC users deposit the biowaste together with the crushed vegetable waste as BA in the supply module. As more batches of biowaste are deposited, a larger volume of the module is occupied and degradation causes an increase in temperature. Once the supply module is full, the material is transferred to the second module, where the material from the supply module is mixed and watered. The second module can be called a homogenisation module because it is where all the material from the supply module is completely mixed. In the homogenisation module, a darkening of the material occurs and the moisture needs to be controlled, since the material can still reach thermophilic temperatures. After a few weeks, when the temperature falls, the material is transferred to the maturation module where it is kept until the characteristic dark brown compost is obtained. This simply needs to be sieved to allow it to be used as fertiliser or organic soil amendment. In the VF model, users deposit the biowaste and cover it with BA as in the HF model. However, once the modular unit is full, the material remains in the supply unit until the compost is removed, usually requiring more processing time than in the HF model. The municipality’s staff is responsible for mixing the inputs, controlling the process and sieving the compost. In the HF methodology, the staff also transfers the material between the different modular units.

CCC must also have a container or tank next to the supply module to store the crushed plant material used as BA. This material gives porosity to the mixture and allows the biowaste to be covered once it has been deposited by users. A water source is recommended to irrigate the composting material and maintain moisture at close to 60% (Torrijos et al. 2021) but also for cleaning the tools and surfaces of the different parts. The production of leachate and the needs for irrigation during composting make it necessary for CCC to be installed on a porous surface that allows the water to drain and prevents swamping.

### Green waste as BA

The Galician climate is characterised as having rainy seasons. The average rainfall is close to 1200 mm, usually concentrated in the autumn and winter. The high rainfall and steep relief allow the development and growth of a large plant biomass (Calvo de Anta et al. 2020). This implies that the maintenance of green areas by municipal services or other managers is considered a problem rather than an opportunity and, in many cases, the waste is unused or disposed of incorrectly. Green waste is made up of different materials of plant origin such as remains from pruning trees and shrubs, grass, leaves and green plants. In the municipalities, pruning remains and thick branches may be used by residents as an energy resource. As for the remains of underbrush, along with leaf litter and grass clippings, it is usual to burn them without energy recovery, deposit them in the bin for undifferentiated waste and, in some cases, crush and accumulate them for degradation but without process control. The composition of the green waste is variable and depends on the time of year (López et al. 2010) but it is characterised by a high content of recalcitrant components such as lignin, cellulose and hemicellulose (Reyes-Torres et al. 2018; Mishra and Yadav 2021). Seasonal production implies that pruning work is carried out mainly in autumn–winter whilst the largest amounts of grass cuttings are generated in spring and summer with the increase in temperatures. The remains of leaves that are produced in the autumn with leaf-fall, hedge trimmings and the remains of cleared vegetation generated throughout the year are also included. To prevent dumping, burning or undifferentiated disposal of green waste in bins, some Galician municipalities such as Allariz or Mondariz have opted to install green waste collection points (Fig. 2). These places, called green points, are marked-out locations beside roads and in visible areas, which may contain a CCC or be close to an urban garden. Residents leave green household waste there. This waste may be distributed, depending on its type, and employed for various uses such as BA, direct composting and an energy source.

### Analytical methods

The physicochemical and biological characterisation of a total of 46 community compost samples was carried out: 22 samples from the VF model and 24 from the HF model. For each compost sample, several parameters were analysed. Moisture content (drying at 105 °C until constant weight) and organic matter content (ignition at 550 °C in a muffle furnace until constant weight) were analysed according to FCQAO (1994). Compacted bulk density, electrical conductivity (EC) and pH were analysed according to UNE-EN standards (UNE-EN 13,037:2012, 13,038:2012 and 13,040:2007) using a Crison CM 35 conductivity meter and a Crison Basic 20 pH meter. The germination index (GI) was calculated by determining seed germination and root length of *Lepidium sativum* growing in 2 mL of aqueous extracts (1:5, w/v) in Petri dishes lined with a paper filter for 48 h (Zucconi et al. 1981). The self-heating test was carried out in the final compost using a 2 l Dewar flask for 10 days at room temperature (FCQAO 1994). Total nitrogen (TN) content and total carbon (TC) content were determined by combustion of dried samples using a LECO 2000 CN elemental analyser. The total heavy metal and nutrient content was determined, following acid digestion, with ICP-OES and Hg content with CV-AAS in the CACTI laboratory at the University of Vigo. Samples of BA used in CC were taken from 4 different municipalities. Particle size composition was determined gravimetrically using 3 sieves with meshes of different sizes: 2 cm, 1 cm and 0.5 cm.

## Results and discussion

### Composting as biowaste treatment

In recent years, various public institutions in the Galician region have committed to treating the organic fraction in the same municipality as it is produced in (Table 1). This reduces the amount of waste transferred to the centralised plant for biowaste incineration with energy recovery. Municipalities with high population densities (more than 500 inhabitants/km^2^) such as A Coruña, Pontevedra, Vilagarcía de Arousa, Cambados, Illa de Arousa, Poio, Moaña, Cangas, Marín and Redondela are on the Galician coast, whilst inland municipalities have a lower population density. Also, each municipality has a different population distribution with a greater or lesser number of population centres depending, amongst other factors, on its history, orography, transport connections and proximity to resources. The number of CCC implemented in each municipality does not only depend on population density or the number of possible suitable areas for CCC but also depends on political decisions. The implementation of CC in these municipalities is usually accompanied by the promotion of HC and citizen training. In order to achieve successful decentralised composting, the involvement of the population and the supply of on-site composting systems by local or regional bodies are necessary. Performing awareness-raising campaigns to educate and train citizens about composting, biowaste separation and correct operations, etc. is a necessary practice to implement local on-site composting (Dri et al. 2018). Carrying out these new measures produces decentralisation of the waste collection and treatment service and reduces the environmental impact by opting for treatment at source via home and community composting. Adhikari et al. (2010) showed that home and community composting reduces greenhouse gas emissions compared with landfill because the emissions produced during the collection and transport of biowaste are removed.

**Table 1:** Municipalities in Galicia with community composting implemented. Population data for 2021, area and the number of CCC in each municipality are included. Data of CCC obtained from Santiago de Compostela (2022) and Pontevedra Provincial Council (2022)

Municipalities	Population (2021)	Area (km^2^)	No CCCs	Municipalities	Population (2021)	Area (km^2^)	No CCCs
< 5000 population				10,000–300,000 population			
Mondariz-Balneario	675	2.3	10	Foz	10,078	100.3	1
Pobra do brollón	1615	176.7	6	Grove, O	10,699	21.9	10
Campo Lameiro	1769	63.8	1	Bueu	11,987	30.8	4
Lama, A	2410	111.8	3	Baiona	12,286	34.5	4
Portas	2860	22.7	1	Cambados	13,673	23.4	5
Oia	3104	83.3	2	Tomiño	13,730	106.6	15
Lousame	3235	93.7	3	Gondomar	14,920	74.5	2
Barro	3622	37.6	1	Poio	17,230	33.9	4
As Neves	3770	65.8	5	Tui	17,398	68.3	5
Mondariz	4394	82.3	12	Sanxenxo	17,635	45.1	3
Cuntis	4643	79.8	1	Moaña	19,496	35.1	11
Meis	4796	52.4	1	Lalín	20,199	326.8	1
Illa de Arousa, A	4951	6.9	5	Porriño, O	20,212	61.2	1
5000–10,000 population				Ponteareas	22,942	125.5	2
Ribadumia	5157	19.7	2	Marín	24,248	36.7	2
Cerdedo-Cotobade	5719	213.3	1	Cangas	26,708	38.1	6
Valga	5768	40.6	3	Ribeira	26,839	68.8	2
Vilaboa	5955	36.9	11	Redondela	29,192	52.1	1
Allariz	6314	86.0	24	Vilagarcía de Arousa	37,545	44.2	2
Rosal, O	6376	44.1	3	Pontevedra	83,114	118.5	33
Soutomaior	7482	24.9	1	Santiago de Compostela	97,858	220.1	3
Salceda de Caselas	9249	35.9	5	A Coruña	245,468	37.8	1
Caldas de Reis	9788	68.3	4				

In the scientific literature, there are different decentralised composting experiences with different types of infrastructure and participants (Villar et al. 2017; Mato et al. 2019; Weidner et al. 2020). Villar et al. (2017) described how the composting of organic fraction waste is carried out in the municipality of Allariz (Spain). This municipality chose HC in homes with garden and CC in two forms: CCC distributed in the urban and periurban centre of the municipality and a dynamic bioreactor for large producers such as catering establishments and food companies. This type of rotary drum was also used to treat waste from a residential community in Dublin in the publication by O'Sullivan and Curran (2011). Other systems that can be used for large producers are forced-ventilation reactors, as shown by Kliopova et al. (2019) in the composting of waste from a catering company or low-technology and low-budget systems such as pile composting (Keng et al. 2020). The compost obtained through these systems is applied to local soils and crops, such as urban gardens, achieving the development of self-consumption and local commerce, with a commitment to the circular recycling of organic matter.

### CCC distribution

In plans to implement CC, it is important to consider the different factors that make it possible to select the points where the CCC are going to be installed. CCC distribution can be analysed using geographic information software (Pai et al. 2019; Colvero et al. 2020) taking into account the location of the houses and the existence of plots of suitable characteristics and size for the CCC. Georeferenced information on the location of homes and the number of inhabitants per home can be obtained from instruments such as the local population census and the cartographic land register. Besides proximity to biowaste producers, CCC plot placement must meet other conditions. As mentioned above, a slightly sloping plot with adequate connections and access for citizens as well as the availability of water sources are desirable conditions for a plot intended for CC (Fertile Auro 2019; Mato et al. 2019). Closeness to urban gardens and green waste deposit points are also good determining factors for municipalities to install a CCC.

**Fig. 3:**
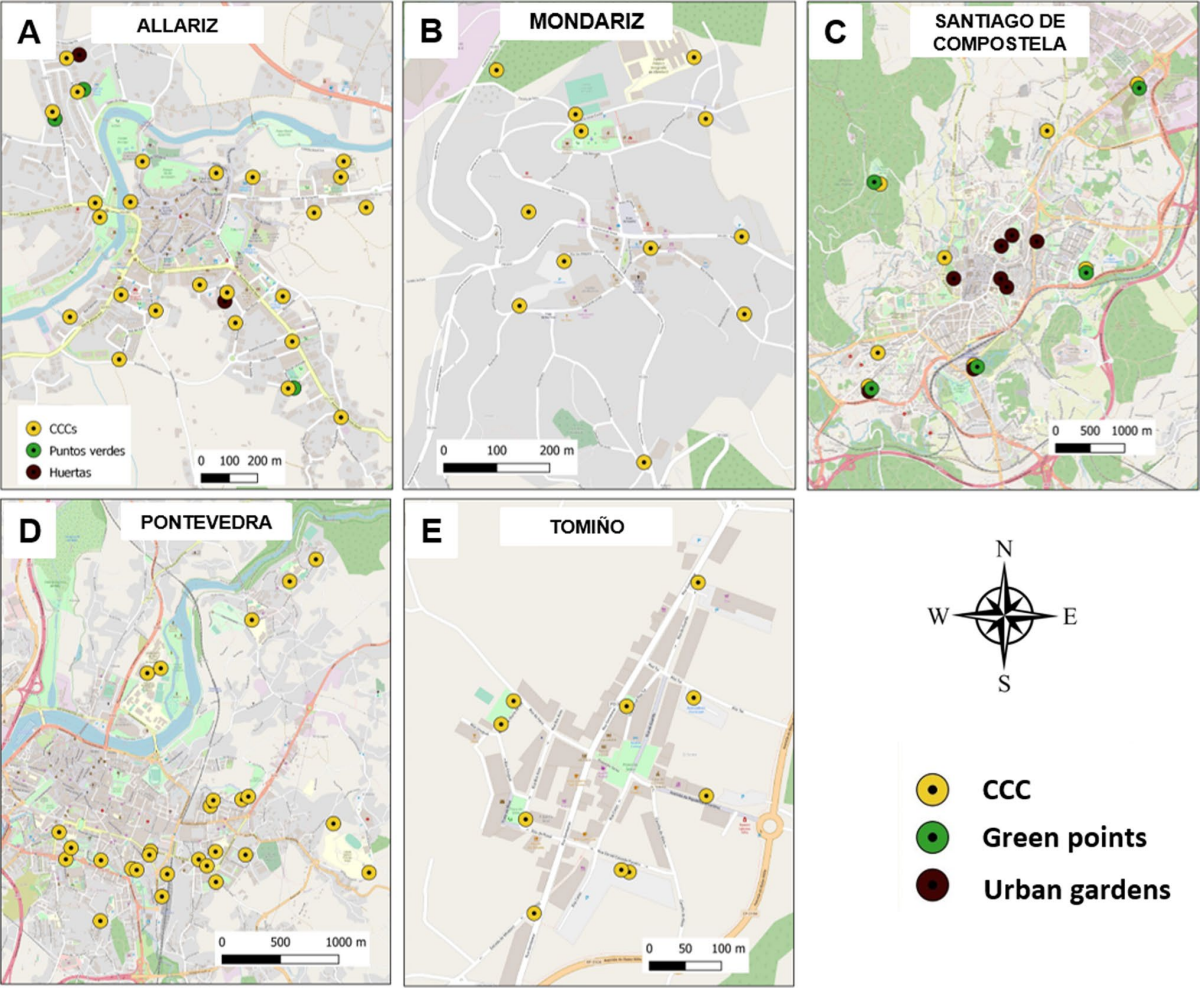
Distribution of CCC (yellow circle), green points (green circle) and urban gardens (brown circle) in the municipalities of **a** Allariz, **b** Mondariz, **c** Santiago de Compostela, **d** Pontevedra and **e** Tomiño. Data obtained from Santiago de Compostela (2022) and Pontevedra Provincial Council (2022)

Figure 3 shows the distribution of the CCC, green points and urban gardens in the municipalities of Allariz, Mondariz, Santiago de Compostela, Pontevedra and Tomiño. The city councils have a central district with old urban planning where it is very difficult to find suitable locations to carry out CC. These areas are highly populated and often lack nearby parks or gardens large enough to accommodate the installation of a CCC. Separate collection of biowaste by means of a bin with a key (Pontevedra, Fig. 3d) or door to door (Allariz, Fig. 3a) is usually one of the best strategies. Intermediate districts have a high population density but more recent urban planning that includes green areas where CCC can potentially be established and where HC does not make sense since most of the homes are in multi-family buildings. In the most peripheral neighbourhoods in the municipality, made up of houses with gardens and orchards, HC and green points for pruning remains collection are appropriate. The size of these districts depends on the population distribution. There are municipalities with fewer than 5000 inhabitants (Mondariz) and municipalities with more than 80,000 inhabitants (Pontevedra). In turn, municipalities can have several separate CCC zones defined by the existence of population centres in the outskirts (Pontevedra, Fig. 3d). Municipalities such as Tomiño (Fig. 3e) with recent urban planning, adapted to the expansion of the municipality, have abundant green and community areas close to the centre of the population nucleus. This condition increases, to a large extent, the area allocated to CC and reduces the door-to-door separate collection area for biowaste.

CC seeks to make the best use of biowaste generated in various homes or activities involving several members from a community in a common project and with follow-up carried out by volunteers or municipal workers. This type of composting adds the social component to HC, associated with the fact of sharing a space, an activity and the interest in collaborating with the conservation of the immediately surrounding environment (Yaben 2012). Marcello et al. (2021) concluded that the residents of three municipalities in the province of Siena, Tuscany (Italy), positively valued the change from centralised recycling to local composting. CC and, in general, decentralised composting stimulate the local economy by creating small businesses at the local level (Pai et al. 2019). However, Weidner et al. (2020) point out that it is necessary to improve user training and provide the necessary means to allow composting to be carried out in adequate conditions.

### Community composting bulking agent

Green waste can originate from both private and public gardening as well as from the forestry and agricultural sectors. Such waste is characterised by a high carbon content and requires a longer degradation process time due to the more recalcitrant components such as lignin, cellulose and hemicellulose present in variable amounts (Mishra and Yadav 2021). Estimates of green waste production in climates similar to Galicia can be found in the scientific literature. Eades et al. (2020) estimated the generation of green waste in the Test Valley Borough Council district in Hampshire (UK) and concluded that the amount generated by rural households was 1.96 ± 1.35 kg per day and 0.64 ± 0.46 kg per day for urban households. Considering these data, the annual production of green waste in a rural municipality of 5000 inhabitants may be around 3500 tonnes/year of green waste. For many years, green resources have been used as an energy source (Hla and Roberts 2015), as a food resource for livestock or as fertiliser for farmland. However, abandonment of grazing in the Galician mountains, and of agricultural and livestock farming, has contributed significantly to the increase in waste of vegetable origin. The Galician forest has gone from being a resource to becoming an environmental problem due to the increase in temperatures and lack of rainfall in an increasingly long fire season (Vega et al. 2009). There is a great deal of plant-based waste in the Galician Community due to the weather and the fertility of the soil. According to Macías et al. (2004), Galician soils have a high potential biomass production capacity with wood production values amongst the highest in Europe. The relief and topography have an important influence on soil organic matter. Variations in altitude, orientation and insolation as a result of the topography influence the spatial distribution of materials and water, creating microclimates in certain local areas. Taking these conditions into account, Calvo de Anta et al. (2020) point out that the Galician Community is the Spanish region with the highest content of organic carbon in the first few centimetres of the soil.

All this regional plant biomass generated can be used for direct composting or can be used as BA (Reyes-Torres et al. 2018). The use of this waste as co-substrate improves and accelerates the composting of other waste (Bustamante et al. 2016), whilst reducing greenhouse gas emissions (Morales et al. 2016) and contributing to lowering the content of micropollutants. However, pretreatment is required for it to be used as BA (Reyes-Torres et al. 2018).

Chang and Chen (2010) demonstrated the effects of mixing biowaste with a material that provides porosity. Providing an adequate FAS for the composting of kitchen waste improves the aerobic conditions of the biowaste. However, porosity is not the only intrinsic property required of a good BA. Others include its capacity to capture and/or transfer water depending on the needs of the process (Chang and Chen 2010). The complexity and heterogeneity of the biowaste deposited by citizens in the CCC make it difficult to obtain specific moisture for these materials. The starting moisture of biowaste can be around 60–80% and can reach values above 90% (Arrigoni et al. 2018; Keng et al. 2020). Due to the fact that the biowaste will change its density and moisture with each contribution by the participants, it is considered necessary to focus on the characteristics of the BA, using appropriate equipment and tools that provide the best conditions and allow a mixture with 40–60% moisture (Manu et al. 2019). In short, the physical, chemical and biological characteristics of the BA are decisive and all of them will influence the composting process to a greater or lesser extent.

**Fig. 4:**
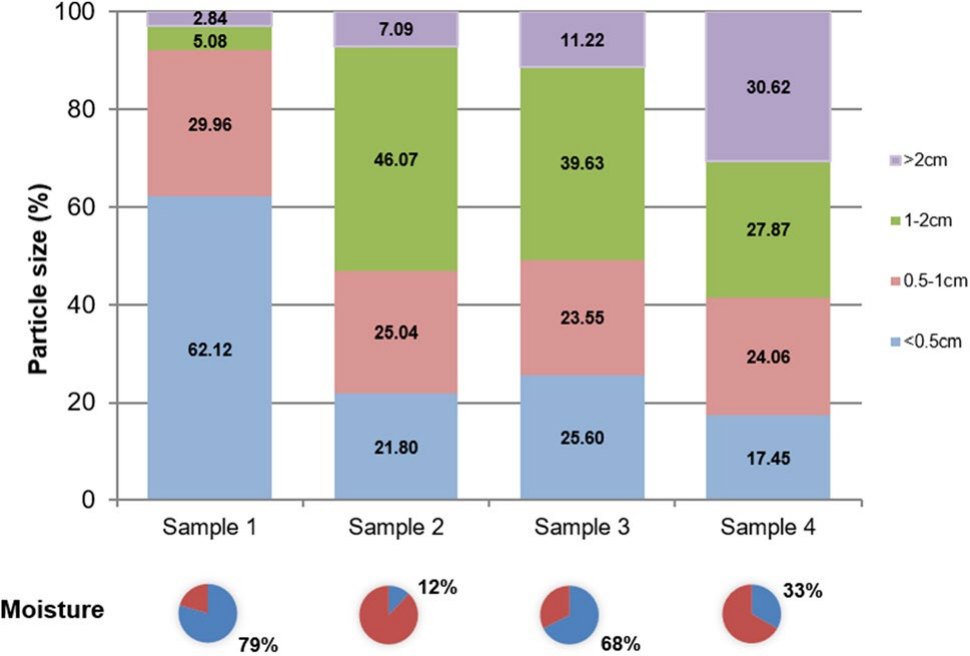
Distribution of the particle size of the four bulking agents in a bar graph and moisture in the sector diagram

Several papers characterise different BA based on the parameters of pH, density and C/N ratio (Schwalb et al. 2011; Li et al. 2013). In the present research, the BA used in the CCC in four different municipalities was analysed. Fresh weight densities of 114 kg/m^3^, 52 kg/m^3^, 59 kg/m^3^ and 34 kg/m^3^, respectively, were obtained for samples 1, 2, 3 and 4 (Fig. 4). Sample 1 was made up of more than 90% of particles smaller than 1 cm, and its moisture was close to 79%, which did not allow the collection of excess water from food remains. Sample 1 also had a high density, close to the density of the biowaste, which gave it a low capacity as a BA considering the volumes treated in the CCC. Samples 2 and 3 had very similar particle size percentages, with the 1–2 cm fraction being the most abundant and with densities similar to those found by other authors (Wang et al. 2022). The sum of the 0.5–1 cm and 1–2 cm fractions accounted for more than 50% by weight in samples 2, 3 and 4. This fraction (0.5–2 cm) stands out from the others because, on the one hand, it provides the mixture with greater porosity without affecting the temperature increase and, on the other hand, a considerable part of this fraction can be recovered for the next composting cycle. The low moisture content of sample 2 (12%), similar to the data provided by Chang and Chen (2010) for sawdust, can cause the emission of finer particles into the air, causing discomfort to those who handle the BA, but it corrects the excess moisture of the biowaste. The granulometric fractions of sample 4 were fairly balanced. The largest fraction consisted of sizes greater than 2 cm, accounting for 30% of the analysed sample, but with values close to the following two fractions. The coarser fractions have the advantage of being recovered, for the most part, after passing through the sieve and, subsequently, recirculating for a new composting cycle (López et al. 2010) and they can act as accelerators of the process as a microbial inoculum. The density is similar to that found by Iqbal et al. (2010) for sawdust because the fraction greater than 2 cm is usually composed of leaves and other light materials. The moisture content (33%) was appropriate for this system because it allows the excess moisture to be absorbed without causing the displacement of the finer particles present in the BA.

### Compost obtained in CC

**Fig. 5:**
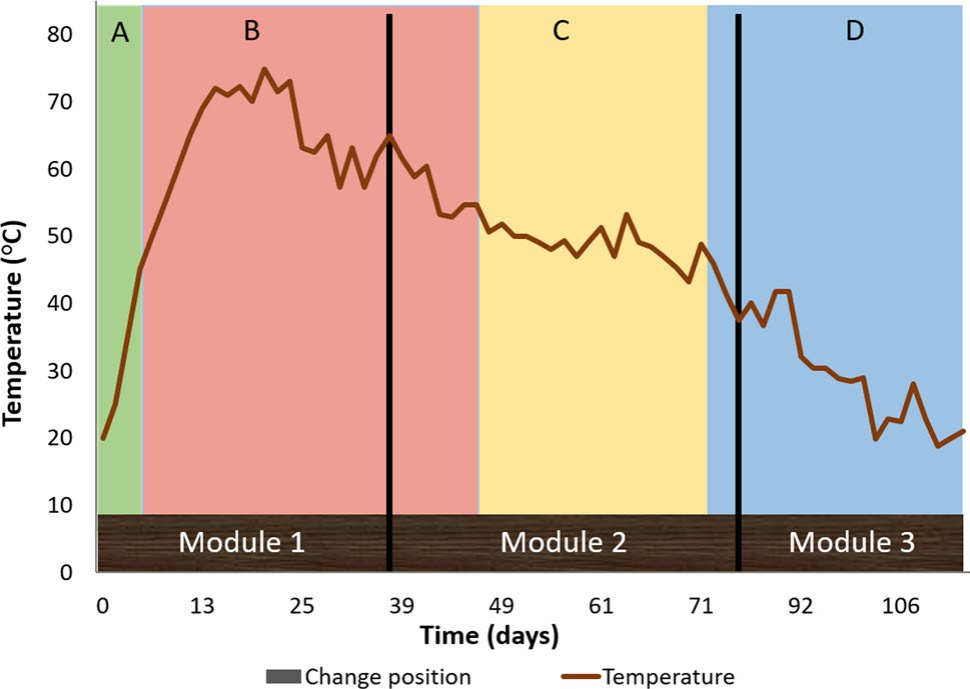
Temperature evolution in a CCC with horizontal flow (HF). Composting phases: activation (**A**), thermophilic (**B**), cooling (**C**) and maturation (**D**)

Figure 5 shows the typical temperature profile of a CCC with the HF model in which the material first passes through the contribution module (module 1), then the homogenisation module (module 2) and finally the maturation module (module 3) (Mato et al. 2019). The temperature profile shows an evolution in accordance with a typical composting process, reaching thermophilic temperatures (> 45 °C) in the contribution module. The thermophilic temperature values continue once the transfer to the homogenisation module had been carried out. The transfer of the material between the modules avoids the compaction and stratification of the material that can occur in vertical flow composters (Arrigoni et al. 2018). The temperatures reached depend on the type of biowaste provided and the environmental temperature where the CCC is located. The presence of animal protein remains in biowaste produces greater increases in temperature that accelerate compost maturation (Storino et al. 2016). Temperature changes in modules 2 and 3 come from the reactivation of the material by turning it over, homogenising it and watering it; however, this reactivation does not usually go beyond 30 °C (Mato et al. 2019). Through the transfer system, the effective capacity of the CCC increases and each modular composting unit is assigned to different phases of the process in independent spaces, so the specific parameters required for each phase can be individually monitored (Fertile Auro 2019). Christensen et al. (2002) studied how the separation of the composting process reduces the possibilities of contamination of mature compost with waste at source and facilitates the detection of biological contamination in the different phases. The HF model allows the material to be homogenised and humidified more efficiently than the VF model because the transfer facilitates these tasks and this provides better use of the capacities of the composting modules.

The compost generated in the CCC were analysed before being applied to the soil to determine its quality and its state of maturity. The data for the 22 samples of compost from the CCC with the VF model and the 24 samples of the CCC with the HF model are shown in Table 2, together with the thresholds for fertiliser products according to Spanish standards (BOE 2013), as well as parameters of special importance to determine their stability and maturation.

**Table 2:** Analytical results of the compost obtained in the CCC with the VF (vertical flow) and HF (horizontal flow) treatment models

	VF	HF	Threshold
Moisture (%)	59.7 ± 11.4 a	69.5 ± 5.4 a	< 40*
Organic matter (%)	63.3 ± 14.2 a	76.8 ± 6.5 a	> 35*
Compacted bulk density (g·L^−1^)	423.8 ± 53.8 a	453.9 ± 37.4 a	–
pH	7.7 ± 0.3 a	7.6 ± 0.4 a	Requirement*
Electrical conductivity (mS cm^−1^)	1.1 ± 0.5 a	0.7 ± 0.2 a	Requirement*
Total carbon (%)	31.2 ± 7.1 a	35.6 ± 2.9 a	–
Total nitrogen (%)	2.3 ± 0.6 a	2.6 ± 0.5 a	If it exceeds 1%*
C/N ratio	13.6 ± 3.3 a	15.1 ± 8.1 a	< 20
CaO (%)	6.5 ± 2.8 a	5.9 ± 1.4 a	–
K_2_O (%)	1.6 ± 0.5 a	1.3 ± 0.2 a	If it exceeds 1%*
MgO (%)	0.5 ± 0.1 a	0.6 ± 0.3 a	–
P_2_O_5_ (%)	1.4 ± 0.5 a	1.2 ± 0.3 a	If it exceeds 1%*
FeO (%)	0.5 ± 0.2 a	0.4 ± 0.1 a	–
Self-heating test	IV–V	IV–V	–
Germination index (%)	88.6 ± 15.9 a	98.5 ± 16.3 a	> 80
*Salmonella* spp. (in 25 g)	Absence	Absence	Absence*
*Escherichia coli* (ufc/g)	100% < 1000	92% < 1000	< 1000*

Values with the same letter are not significantly different (paired-sample Student’s t-test, $p < 0.05$)

*Regulation proposal for organic fertilisers in Spain (BOE 2013)

All the samples analysed had a percentage of less than 0.1% of inert materials larger than 2 mm, such as plastics or glass, due to the quality of the starting waste and the BA used. The training of the participants and detection of improper items whilst turning the material are usually the work of the technician responsible for the CCC. The presence of a person with training in composting is essential to communicate and train the participants on how to separate their waste (Fertile Auro 2019).

In relation to pH, the values were consistent with other publications for the composting of this type of waste (Keng et al. 2020). Li et al. (2013) established the optimal pH values of the final compost at 7 to 8. However, some biowaste such as citrus fruits may have a very acidic pH and it is necessary to add some additives, such as ash, to maintain the pH at the desired level and shorten the composting time to improve the efficiency of the process. The EC is decisive for the final application of the compost to the soil since a high content of this parameter can cause adverse effects on the germination and growth of the seeds (Komilis and Tziouvaras 2009). However, the EC was slightly lower than that shown by other researchers (Moldes et al. 2007; Mishra and Yadav 2021) and no negative effect was observed on the germination rate (86% and 98.5%). Irrigation during the process can reduce the salt content through the leachate but also the different proportion of precooked and postcooked starting waste, the latter with higher salinity. Regarding the parameters of maturity and stability analysed, they indicated that the compost obtained reached an optimal maturity to be applied to the soil as an organic amendment. The organic matter mineralises during the composting process but remained in percentages much higher than 35% (BOE 2013) in all compost samples. The organic matter showed stability conditions indicated by the self-heating test (IV–V) and the C/N ratio (13.6 ± 3.3 and 15.1 ± 8.1) similar to other values shown in the literature for biowaste compost (Morales et al. 2016; Storino et al. 2016). Regarding the germination test, more than 81% and 83% of the samples reached germination values higher than 80% for the VF and HF treatments, respectively. This demonstrated a high degree of maturity of these products, and none of the samples of the two treatments had values lower than 50% established by Zucconi et al. (1981) as an indicator of high plant phytotoxicity.

Microbial analysis (*Salmonella spp.; Escherichia coli*) indicated the sanitisation of most of the compost. This shows that thermophilic temperatures were maintained long enough to remove the pathogen load. In this respect, 92% of the HF treatment samples and all of the VF treatment samples had values in accordance with the standard. *E. coli* contamination of some HF treatment samples may be due to short treatment processes, composting time being one of the key factors in reducing pathogen load (Storino et al. 2016). On the other hand, the total nutrient content determines the fertilisation potential of the compost. Moldes et al. (2007) stated that compost produced from biowaste has a high proportion of nutrients (P, Mg, K and Ca) and these macronutrients can reach higher values than other substrates such as peat, containing amounts necessary for plant growth. Vázquez and Soto (2017) found percentages of Ca and Mg close to the present study in compost obtained from domestic composting. However, the mean values of K and P were higher and lower, respectively, than those detected in the CCC. Instead, Keng et al. (2020) obtained lower and higher percentages of K and P, respectively, than the compost produced in the CCC in this study. The heterogeneity of the starting biowaste determines the content of nutrients present in the final compost. For this reason, the regulations require them to be stated on the compost label if they are greater than 1%.

**Fig. 6:**
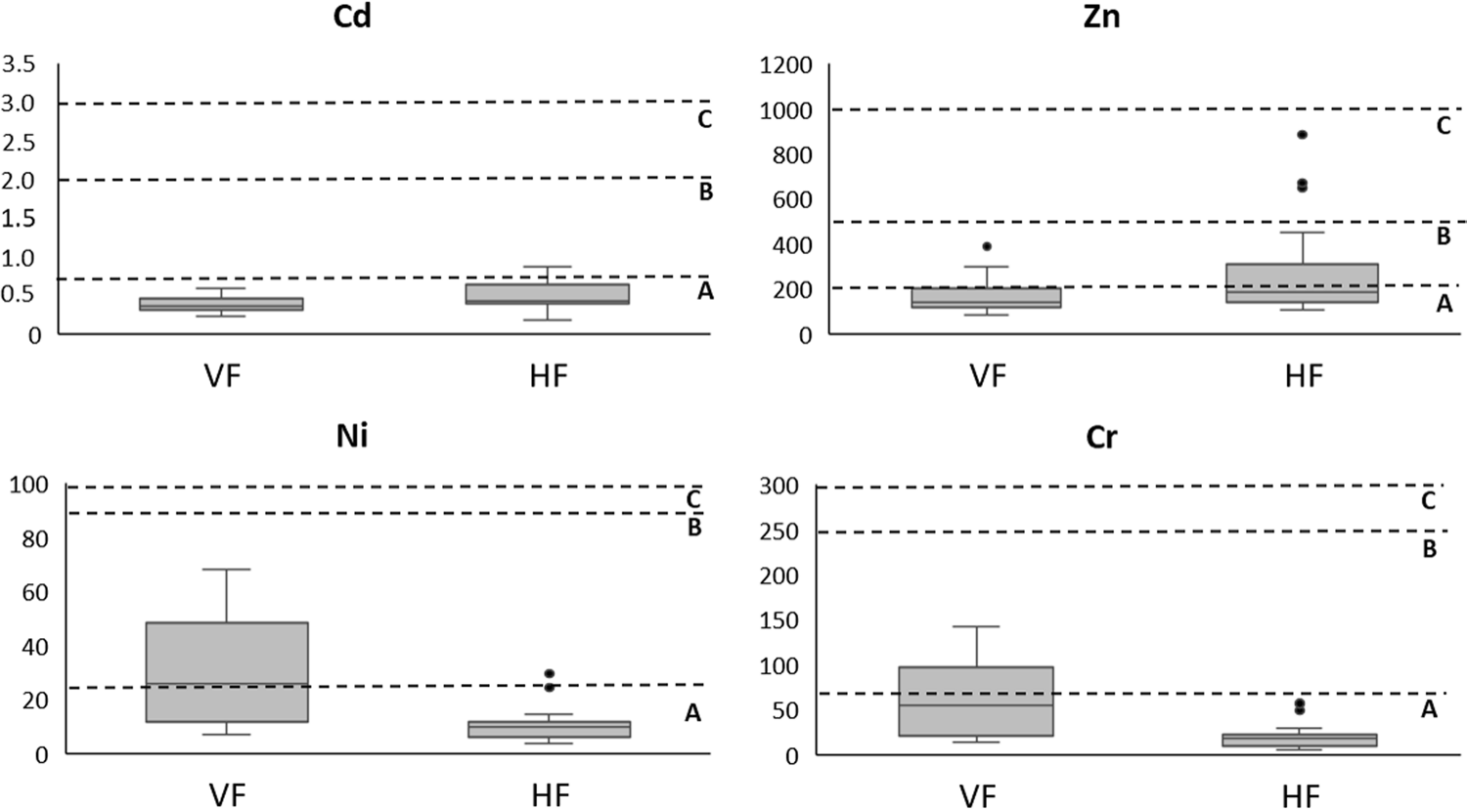
Concentrations of heavy metals Cd, Zn, Ni and Cr present in the compost obtained through the VF (vertical flow) and HF (horizontal flow) treatments. *Outliers

Spanish regulation (BOE 2013) classifies compost into three categories according to its heavy metal content: classes A, B and C. If the metals are considered separately, all the samples belong to class A for Hg (< 0.4 mg kg^−1^ dw), Cu (< 70 mg kg^−1^ dw) and Pb (< 45 mg kg^−1^ dw) (data not shown), as observed in other research (Storino et al. 2016; Manu et al. 2019). Regarding Cd, more than 90% of the samples met the levels for class A (< 0.7 mg kg^−1^ dw). Torrijos et al. (2021) showed similar values for this metal in compost from food waste. The heavy metals Ni, Zn and Cr presented greater differences. Figure 6 shows the variability in the concentration of these metals, classifying the composts in the respective categories and obtaining different values for the two treatments studied. The atypical data observed in Zn correspond to a sample of VF model and different compost samples of the HF model with a metal concentration 2 or 3 times higher than the mean values and, therefore, can be considered point pollution (tools, poorly sorted waste, intrinsic heavy metals in food or the BA source). VF samples had higher values for Ni and Cr while the HF model had atypical values that can be located in concentrations close to class B for Ni. Following the regulations, 58% of the samples were classified as class A compost and 42% of the compost as class B and C. The presence of these heavy metals in the final compost can have different origins, and all metals can be increased to the point of finding contaminated compost that does not reach class C quality (Vázquez and Soto 2017). O'Connor et al. (2022) concluded that the origin of heavy metal contamination in soils and biowaste may be conditioned by the quality of the fertilisers, pesticides and plastics used. To avoid interference of point pollution in heavy metal analysis, it would be necessary to analyse more samples from each batch. However, it is important to note that the compost batches of each CCC are around 500 L, which are considerably smaller after screening for removal of the BA. Therefore, taking more samples from the same batch is neither economically nor operationally feasible. In order to establish the quality of compost from a specific CCC, quality acceptance sampling should be carried out for continuous production by establishing levels and periodically analysing batches of compost.

## Conclusions

The location of the CCC depends on the city council’s urban planning and the presence of community green areas close to areas with multi-storey dwellings. To carry out adequate treatment of the biowaste deposited in the CCC, it is necessary to have a BA with the ideal size and moisture conditions. The most-used particle sizes mixed with biowaste in CCC are between 0.5 and 2 cm in length because they are the ones that best adapt to these treatment volumes (1 m^3^). The particle size composition of the BA should be balanced and its moisture content should be around 30% to provide aeration, capture water from biowaste, facilitate handling by neighbours and adequately cover biowaste inputs.

The two treatment models carried out in the CCC are appropriate to obtain compost with good fertilising properties in accordance with the legal regulations with adequate values of stability and maturity. However, there are differences in effective CCC capacity and material handling in the HF model over the VF model. In contrast, transfer between composting modules requires greater effort and more hours of work in the CCC with the HF model than with the VF model.

In this work, the efficiency of the CC model to manage of biowaste was evaluated. Strategies for CC require a study of the municipality with special attention to urban planning, population distribution and green areas. As the biowaste is of variable composition, it is necessary to characterise the BA used in the CCC to ensure a correct process. The quality of the final product and the real application of CC on a wide territory (extensive) in an integral way (intensive in each municipal territory) was verified.

## Data Availability

The authors confirm that the data supporting the findings of this study are available within the article. Raw data that support the findings of this study are available from corresponding author, upon reasonable request.
